# Sociodemographic and clinical risk factors associated with in-hospital tuberculosis mortality in Türkiye, 2008-2018

**DOI:** 10.5578/tt.202401864

**Published:** 2024-03-26

**Authors:** Mine GAYAF, Merve AYIK TÜRK, Özer ÖZDEMİR, Gülru POLAT, Onur KARAMAN, Filiz GÜLDAVAL, Gülsüm ARI, Dursun TATAR, Ahmet Emin ERBAYCU

**Affiliations:** 1 Clinic of Pulmonology, Health Sciences University, Dr. Suat Seren Chest Diseases and Surgery Training and Research Hospital, İzmir, Türkiye; 2 Clinic of Pulmonology, İzmir Bakırçay University, Çiğli Training and Research Hospital, İzmir, Türkiye; 3 Clinic of Pulmonology, Health Sciences University, İzmir Bozyaka Training and Research Hospital, İzmir, Türkiye

## Abstract

**ABSTRACT**

**
Sociodemographic and clinical risk factors associated with in-hospital tuberculosis mortality in Türkiye, 2008-2018
**

**Introduction:** *Tuberculosis (TB) is an infectious disease that can be fatal if left untreated or poorly treated, and it is associated with many morbidities. Deaths may provide better understanding of the associated factors and help guide interventions to reduce mortality. In this study, it was aimed to reveal some of the features that predict hospital mortality in patients with TB and to present some alarming findings for clinicians.
*

**Materials and Methods:** *Patients who had been hospitalized with the diagno- sis of TB between January 2008 and December 2018 were included and analyzed retrospectively. In-hospital mortality because of any TB disease after the initiation of treatment in patients admitted to the TB Ward and the pri- mary cause of mortality were taken as endpoint.
*

**Results:** *A total of 1321 patients with a mean age of 50.1 years were exam- ined. Total mortality was 39.4% (521 deaths) and 13.1% were in-hospital deaths (173 deaths). Of the deaths, 61.8% (n= 107) occurred during the first month after TB treatment were started. On univariate analysis, age over 48.5 years, Charlson comorbidity index, extension of radiological involvement, hypoalbuminemia and lymphopenia were most predictive variables with higher odds ratios (respectively, p< 0.001 for all).
*

**Conclusion:** *In-hospital tuberculosis disease mortality is related with older age, cavitary or extensive pulmonary involvement, low albumin levels, unem- ployment, cigarette smoking and especially those with concomitant malig- nancy and chronic pulmonary disease.
*

**Key words:** *Tuberculosis; in-hospital mortality; pulmonary involvement; risk factors; morbidity
*

**ÖZ**

**
Türkiye’de hastane içi tüberküloz mortalitesi ile ilişkili sosyodemografik ve klinik risk faktörleri, 2008-2018
**

**Giriş:** *Tüberküloz (TB), tedavi edilmediği veya yetersiz tedavi edildiği takdirde ölümcül olabilen ve birçok morbidite ile ilişkili bulaşı- cı bir hastalıktır. Ölümler, risk faktörlerinin daha iyi anlaşılmasını sağlayabilir ve mortaliteyi azaltmaya yönelik müdahalelere rehberlik edebilir. TB hastalarında hastane mortalitesini öngören bazı özellikleri ortaya çıkarmayı ve klinisyenler için bazı uyarıcı bulguları sunmayı amaçladık.
*

**Materyal ve Metod:** *Ocak 2008 ile Aralık 2018 tarihleri arasında TB tanısıyla hastaneye yatırılan hastalar çalışmaya dahil edilmiş ve retrospektif olarak analiz edilmiştir. TB servisine yatırılan hastalarda, tedaviye başlandıktan sonra herhangi bir TB hastalığı nedeniyle görülen hastane içi mortalite, birincil sonlanım noktası olarak alınmıştır.
*

**Bulgular:** *Toplam 1321 hasta incelendi ve ortalama yaş 50,1 idi. Toplam mortalite %39,4 (521 ölüm) olup, %13,1’i hastane içi ölümlerdi (173 ölüm). Ölümlerin %61,8’i (n= 107) TB tedavisi başlandıktan sonraki ilk ay içinde gerçekleşmiştir. Tek değişkenli ana- lizde; 48,5 yaş üstü, Charlson komorbidite indeksinin yüksekliği, radyolojik tutulumun yaygınlığı, hipoalbüminemi ve lenfopeni daha yüksek odds oranları ile en prediktif değişkenlerdi (sırasıyla, hepsi için p< 0,001).
*

**Sonuç:** *Hastane içi TB mortalitesi, ileri yaş, kaviter veya yaygın pulmoner tutulum, düşük albümin düzeyleri, işsizlik, sigara kullanımı ve özellikle eşlik eden malignite ve kronik pulmoner hastalık mortalite ile ilişkili bulunmuştur.
*

**Anahtar kelimeler:** *Tüberküloz; hastane içi mortalite; pulmoner tutulum; risk faktörleri; morbidite
*

## INTRODUCTION

Tuberculosis (TB) continues to be the deadliest and single-cause contagious disease in the world. It is estimated that approximately a quarter of the world’s population is infected with Mycobacterium tuberculosis. The year 2019 was the last year when the World Health Organization (WHO) published global death estimates by cause, and tuberculosis was the 13th leading cause of death (1). The COVID-19 pandemic replaced TB deaths as the cause of infectious disease deaths in 2020. It was reported that TB death estimates were considered temporary in 2020. According to WHO, it was reported that an estimated 9.9 million people had TB in 2020, the number of deaths was 1.5 million, and this death estimate returned to the level of 2017 (2). TB is a treatable and preventable disease. More than

60 million deaths have been prevented with TB treatment since 2000 (3).

In general, TB does not necessitate hospital admission for its treatment; however, if there are serious symptoms (e.g. shortness of breath and deterioration in a systemic condition), hospitalization may be required. Most TB patients are hospitalized, and in-hospital mortality rates range between 2% and 12%. A great deal of the costs of TB treatment result from hospitalization against the costs of an outpatient (4).

Several predictors have been associated with a greater risk of mortality in TB patients, including poverty, homelessness, alcohol or drug addiction, irregular/inadequate treatment, late diagnosis of the disease, multidrug-resistant TB (MDR-TB), advanced age, human immunodeficiency virus (HIV) infection,

and comorbid disease like diabetes (5-8). Patients who have malignant tumors are immunocompromised and might have unusual clinical manifestations related to delayed diagnosis and high mortality. TB deaths are crucial indicators of the effects of TB control measures, especially in areas with high HIV and TB prevalence in TB program monitoring (5). Data on TB mortality provide us with a better understanding of the factors associated with mortality and help guide interventions to reduce mortality. However, there is uncertainty on the factors associated with in-hospital mortality among patients with pulmonary TB.

We aimed to reveal some of the features that predict hospital mortality in patients with TB and to present some alarming findings for clinicians. Some risk factors are known in TB follow-up, but there is limited literature data systematically investigating these factors. The aim of the study was to determine the risk factors that increase in-hospital mortality in patients diagnosed with TB.

## MATERIALS and METHODS

**Patient Selection**

A retrospective cohort study was executed from January 2008 to December 2018 at a Chest Disease Hospital in İzmir, Türkiye. A total of 1321 patients diagnosed with TB and hospitalized in TB Ward between 01.01.2008 and 31.12.2018 were included.

Inclusion criteria: Patients with positive tuberculosis culture or clinically and histopathologically compatible with tuberculosis, 15 years of age and older, hospitalized and treated for TB disease.

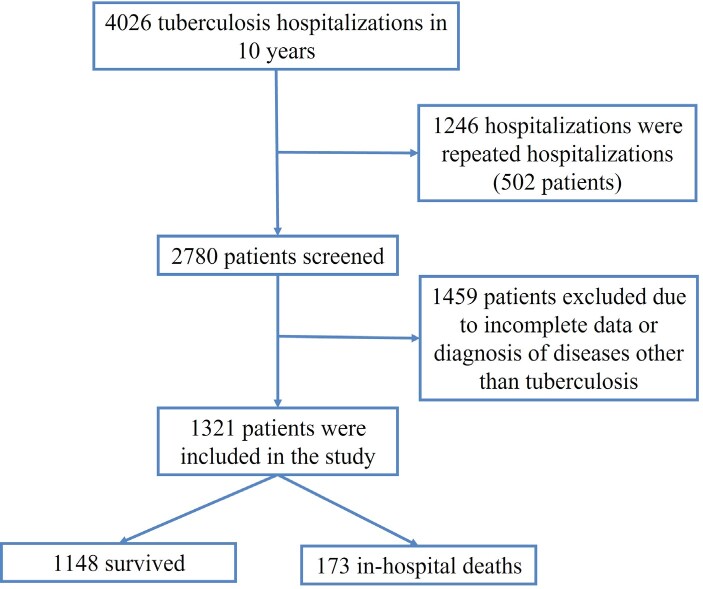

**Figure 1.** Patient disposition chart.

Exclusion criteria: Patients with a diagnosis of atypical mycobacteria without a final diagnosis of TB, who died before TB treatment was started, and those with incomplete microbiological and/or pathological examination (Figure 1).

## Study Design

In-hospital mortality because of any TB after the initiation of treatment of patients admitted to the TB Ward and the primary cause of mortality were taken as endpoint. The survival of the patients (after discharge or treatment) was followed up until 31.12.2021. The sociodemographic data (citizenship status, occupation, social security, whether they lived in a collective place of residence), radiology, laboratory, treatment modalities and morbidities by Charlson comorbidity index (CCI) were collected.

Clinical, laboratory and radiological features of the patients hospitalized with TB were recorded. The characteristics of the mortal group (n= 173) and the non-mortal group (n= 1148) were compared with each other. The comparisons between the main variables of the patients who died during

hospitalization and those who were discharged were calculated statistically.

The latest case definition of the disease, the date of initiation of the treatment, and laboratory values of the patients who had more than one hospitalization over the years were taken into consideration.

Case definitions based on the side of involvement and the history of therapy were classified according to the National Tuberculosis Guide (3);

“Pulmonary tuberculosis” was defined as TB involving the lung parenchyma or the tracheobronchial tree.

“Extrapulmonary tuberculosis” was defined as those with histological and clinical findings consistent with TB or acid-resistant bacillus (ARB) in samples taken from organs other than the lung parenchyma.

“New case” was defined as a patient who did not receive TB treatment before or received treatment for less than one month.

“Drug-resistant TB” was defined according to WHO Global Tuberculosis Program (9).

“Multidrug resistance (MDR)” was defined as resistance to at least both isoniazid and rifampicin.

All bacteriologically confirmed and clinically diagnosed TB cases, except for MDR-TB cases placed on a second-line drug regimen, were treated with four drugs for two months as standard [isoniazid 5

(H) mg/kg, rifampicin (R) 10 mg/kg, ethambutol (E) 20 mg/kg and pyrazinamide (Z) 25 mg/kg]. At the end of the second month, the initial period was extended for one more month with the same drugs in cases with positive sputum smear results. Patients with negative sputum smear results at the end of the

2nd-3rd month were switched to treatment regimen

(HR), and the treatment was completed for a total of six months. Pulmonary and extrapulmonary TB cases were treated with the same treatment regimens. Central nervous system TB was treated for 12 months, and bone TB for nine months (3). Drug-resistant TB was categorized as mentioned above, and treatment was planned according to WHO guidelines (9).

Radiological findings were graded as mild: non- confluent uni- or bilateral lung involvement confined to the apical segment with no visible cavitation; moderate: disseminated uni- or bilateral lung involvement in the absence or presence of cavitation (cavity size <4 cm); or advanced: disseminated uni- or bilateral lung involvement with cavitation (cavity size >4 cm) (10,11).

Case definitions are divided according to the national health department guidelines for bacteriologic diagnosis (3): Smear positive pulmonary tuberculosis and smear negative pulmonary tuberculosis.

Smear-positive pulmonary tuberculosis: patients with ARBs confirmed by smear in at least two sputum samples.

Smear negative pulmonary tuberculosis: Patients with negative sputum smears but positive cultures.

In the histopathologic diagnosis of TB, the presence of granulomatous inflammation, especially necrosis, in the biopsy material taken from any tissue is a histopathologic finding compatible with the diagnosis of TB (3).

Because the study was retrospective in nature, missing data was obtained by calling patients for information that did not exist in the hospital data system.

Patients’ private information was anonymized and their personal health information was protected from disclosure. For better measurement, all data was collected by one researcher. After one researcher collected the data, the second researcher checked them all.

## Statistical Analysis

The analyses were made in SPSS software v22.2 (IBM, NY, USA). The sample size of the study was calculated with the G-power program. The sample size of the study had power as 0.95 and alpha as

0.05. Shapiro-Wilk and Kolmogorov-Smirnov normality tests were used to determine whether continuous data were normally distributed, and Mann-Whitney U and Student’s t-test were used to compare continuous variables along with Chi-square and Fisher’s exact test for the comparison of categorical data. Results were given as mean ± SD, median (min-max), number, and percentage (%). The optimal cut-off values, sensitivity, and specificity values of these parameters were calculated with receiver operating curve (ROC) analysis by using the area under the curve and the Youden index. The predictive values of the parameters for in-hospital mortality were calculated with univariate and multivariate logistic regression analyzes. The results were presented with 95% confidence intervals. P-value <0.05 was considered statistically significant.

## Ethics considerations

This is a retrospective study, and local ethics approval was received with the ethics approval date and number 27-12-2017/8665. All procedures performed in studies involving human participants were carried out following the ethics standards of the institutional and/or national research committee and with the 1964 Helsinki declaration and its later amendments or comparable ethics standards. Informed consent was waived because of the retrospective nature of the study.

## RESULTS

The study population was composed of 1321 patients hospitalized with the diagnosis of TB. Mean age of the patients was 50.1 ± 18.4 years, and there were 954 males (72.2%). Patients mostly had normal body mass index (BMI) (n= 766, 58%). Of the patients, 43% were unemployed and 16.6% did not have any

**Table:** 

**Table 1.** Demographic characteristics of the study population	
**Demographic characteristics**	
Age, years, mean ± SD	50.1 ± 18.4
Sex, male, n (%)	954 (72.2)
Body mass index, kg/m2, mean ± SD, n (%)	21.4 ± 3.9
Underweight (≤18.5)	309 (23.4)
Normal range (18.5-24.9)	766 (58.0)
Overweight (25.0-29.9)	172 (13.0)
Obese (≥30.0)	41 (3.1)
Unknown	33 (%2.5)
Citizenship status, n (%)	
Citizen	1264 (95.6)
Non-citizen	57 (%4.3)
Living environment, n (%)	
Own house	1196 (90.5)
Nursing home	9 (0.7)
Homeless	36 (2.7)
Military personnel	49 (3.7)
Prisoner	31 (2.3)
Employment, n (%)	
Employed	391 (29.6)
Retired	267 (20.2)
Unemployed	568 (43.0)
Student	22 (1.7)
Unknown	73 (5.5)
Presence of social insurance, n (%)	
Present	856 (64,8)
None	219 (16.6)
Green card	246 (18.6)
Education, n (%)	
Uneducated	252 (19.1)
Primary school	863 (65.3)
High school	123 (9.3)
University	29 (2.2)
Unknown	54 (4.1)

social insurance, while 18.6% had a green card, which is provided to the poor and uninsured citizens to provide them free health services. The whole demographic characteristics of the study population are shown in Table 1.

Tuberculosis diagnosis was obtained by bacteriological confirmation in 1170 (88.6%). Smear-positive patients constituted 75% (n= 991), and it was negative in 21.7%. Culture positivity was 85.3% (n= 1127). Of the patients, 86.4% had a diagnosis of pulmonary TB and 4.4% had both pulmonary and extrapulmonary TB and 2.6% had miliary TB. Mostly involved site for extrapulmonary tuberculosis was the pleura (n= 112, 60.9%) following tuberculous lymphadenitis in 31 (16.8%). New patients were the biggest group (87.4%, n= 1154); while the remaining 176 (13.3%) were previously treated patients. Among the previously treated patients, 7.7% (n= 102) were relapse of the disease. There was resistance to at least one of the first-line drugs in 147 (11.1%).

Total mortality was 39.4%, while in-hospital mortality was 13.1%. Mean hospital admissions for an individual patient were 1.3 ± 0.9. Clinical characteristics of the study population are shown in Table 2.

When patients with TB who died in hospital were compared with those who were still alive; older age (p< 0.001), being homeless or in care (<0.001), shorter hospital stay (0.002) and lack of education (0.001) were found to be risk factors.

Although mean BMI of both subgroups was within the limits of normal weight, BMI was lower in in-hospital death group (p< 0.001). Overweight and obese patients were more in the surviving group (p= 0.002).

Tuberculosis of military members, prisoners and employed patients were higher in the surviving group (p< 0.001). Charlson comorbidity index was higher in in-hospital death group (p< 0.001). In the sub- analysis of comorbidities, the presence of chronic lung disease, chronic renal failure, cardiovascular disease, cancer, dementia, and cerebrovascular disease was significantly higher in TB patients who died in hospital (Table 3). Treatment other than HRZE was more in in-hospital death group (p< 0.001). It made no difference for previously treated cases (p= 0.719) or those with extrapulmonary TB (p= 0.85). There were a total of 57 MDR cases, five of whom (8%) died while hospitalized (p> 0.05).

**Table:** 

**Table 2.** Clinical characteristics of the study population (continue)	
Treatment regimen*, n (%)	
HRZS	29 (2.2)
HRZE	1048 (79.3)
Other	244 (18.5)
Completion of the treatment regimen, n (%)	
Complete	1019 (77.1)
Incomplete	181 (13.8)
Unknown	121 (9.2)
Hospital stay duration, mean ± SD (days)	39.4 ± 55.5
Survival, n (%)	
Deaths	521 (39.4)
In-hospital deaths	173 (13.1)
Timing of in-hospital deaths, (from the	
initiation of treatment), n (%)	
The first month	107 (61.8)
1-4 months	42 (24.3)
4-12 monthsÝ	24 (13.9)
* H: Isoniaside, R: Rifampisin, Z: Pyrazinamide, E: Ethambutol, S: Streptomycin, Ý: Deaths reported at 4-12 months are also in-hospi- tal deaths.

In the analysis of radiological involvement and laboratory, common extensive (p< 0.001), bilateral (p< 0.001) and cavitary (p= 0.004) pulmonary involvement, lower protein, albumin, hemoglobin, lymphocytes, bacterial culture positivity (p< 0.001) and higher neutrophils (p= 0.006), neutrophil to lymphocyte ratio (p< 0.001) were revealed in in-hospital death group (Table 4).

Cut-off values with the best sensitivity and specificity values were determined for age, CCI, hospital stay duration, and laboratory parameters (protein, albumin, hemoglobin, lymphocyte, and neutrophil) for univariate and multivariate logistic regression analysis. On univariate analysis, age over 48.5 years, CCI, extension of radiological involvement, hypoalbuminemia and lymphopenia were most predictive variables with higher odds ratios (p< 0.001 for al). On multivariate analysis, extension of radiological involvement was associated with higher in-hospital mortality (OR= 3.98, 95% CI, 1.48-10.76, p= 0.006). Also,

hypoalbuminemia, neutrophilia, CCI, cavitary disease, age over 48.5 years and lymphopenia were other significant factors that are predictive of higher in-hospital mortality (respectively, p< 0.001; p< 0.001; p= 0.001; p< 0.001; p= 0.006; p= 0.04) (Table 5).

**Table:** 

**Table 3.** Comparison of demographic and clinical parameters of the survivors and in-hospital deaths			
**Variables**	**In-hospital deaths (n= 173)**	**Survived (n= 1148)**	**p**
Age, years	64.0 (24.0-91.0)	49.0 (15.0-90.0)	**<0.001**
Sex, male, n (%)	130 (75.1)	824 (71.8)	0.36
Body mass index, kg/m2	19.5 (12.1-34.7)	21.3 (12.8-46.8)	**<0.001**
Body mass index, group, n (%)			
Underweight (≤18.5)	59 (34.7)	250 (22.4)	
Normal (18.5-24.9)	93 (54.7)	673 (60.2)	
			**0.002**
Overweight (25.0-29.9)	16 (9.4)	156 (14.0)	
Obese (≥30,0)	2 (1.2)	39 (3.5)	
Citizenship, TR, n (%)	169 (97.7)	1095 (95.5)	0.19
Hospital admission, n (%)	1.0 (1.0-7.0)	1.0 (1.0-12.0)	0.059
Hospital stay duration, days	19.0 (1.0-206.8)	21.0 (1.0-800.0)	**0.002**
Living environment, n (%)			
Own house	151 (87.3)	1045 (91.2)	
Nursing home	5 (2.9)	4 (0.3)	
Homeless	16 (9.2)	20 (1.7)	**<0.001**
Military personnel	0 (0.0)	49 (4.3)	
Prisoner	1 (0.6)	30 (2.6)	
Employment, n (%)			
Employed	48 (27.7)	219 (19.1)	
Retired	17 (9.8)	374 (32.6)	
Unemployed	76 (43.9)	492 (42.9)	**<0.001**
Student	1 (0.6)	21 (1.8)	
Unknown	31 (17.9)	42 (3.7)	
Presence of social insurance, n (%)	124 (71.7)	732 (63.8)	0.05
Uneducated, n (%)	157 (95.7)	958 (86.9)	**0.001**
Smoking, n (%)	116 (67.1)	674 (58.7)	**0.04**
Alcohol usage, n (%)	20 (11.6)	88 (7.7)	0.08
Substance abuse, n (%)	1 (0.6)	12 (1.0)	1.000
Charlson comorbidity index	5.0 (0.0-10.0)	1.0 (0.0-12.0)	**<0.001**
Comorbidity, n (%)	150 (86.7)	578 (50.3)	**<0.001**
Diabetes mellitus	31 (17.9)	216 (18.8)	0.78
Chronic pulmonary disease	84 (48.6)	242 (21.1)	**<0.001**
Liver disease	5 (2.9)	23 (2.0)	0.40
Chronic renal failure	18 (10.4)	33 (2.9)	**<0.001**
Cardiovascular disease	46 (26.6)	130 (11.3)	**<0.001**
Cancer	33 (19.1)	50 (4.4)	**<0.001**
Psychiatric disorders	8 (4.6)	60 (5.2)	0.74
Cerebrovascular disease	23 (13.3)	37 (3.2)	**<0.001**
Dementia	21 (12.7)	14 (1.2)	**<0.001**

**Table:** 

**Table 4.** Comparison of radiological and laboratory parameters of the survivors and in-hospital deaths			
**Variables**	**In-hospital deaths (n= 173)**	**Survived (n= 1148)**	**p**
Radiology, n (%)			
Pleural disease complication	9 (5.2)	38 (3.3)	0.60
Extrapulmonary disease	14 (8.1)	106(9.2)	0.85
Miliary tuberculosis	9 (5.2)	24 (2.0)	0.07
New cases	153 (87.9)	1001 (87.2)	0.72
Extensive disease	105 (66)	258 (24.8)	**<0.001**
Bilateral involvement	124 (78)	538 (51.9)	**<0.001**
Cavitary disease	110 (69.9)	582 (56.1)	**0.004**
Microbiology, n (%)			
Smear positivity	126 (72.8)	867 (75.5)	0.43
Culture positivity	142 (82.1)	989 (86.1)	0.21
Drug resistance	18 (10.8)	129 (11.4)	0.95
Laboratory, median (min-max)			
Protein, g/dL,	6.0 (3.4-8.8)	7.1 (3.4-9.3)	**<0.001**
Albumin, g/dL	2.5 (1.0-4.1)	3.4 (1.0-5.3)	**<0.001**
Globulin, g/dL	3.5 (1.8-5.6)	3.6 (1.1-6.8)	0.18
Hemoglobin, g/dL	10.4 (6.3-15.6)	12.0 (4.8-18.7)	**<0.001**
Lymphocytes, /mm3	0.9 (0.1-23.4)	1.5 (0.1-18.1)	**<0.001**
Lymphocytes, %	9.9 (1.5-79.4)	17.8 (1.2-64.0)	**<0.001**
Neutrophils, /mm3	7.1 (0.1-30.2)	6.0 (0.2-47.0)	**0.006**
Neutrophils, %	81.4 (15.0-97.2)	70.0 (0.5-97.3)	**<0.001**
Neutrophil to lymphocyte ratio	7.9 (0.2-60.0)	4.2 (0.1-65.1)	**<0.001**
Bacterial culture positivity, n (%)	32 (18.5)	37 (3.3)	**<0.001**

## DISCUSSION

The best predictive factors of in-hospital tuberculosis mortality were older age, being homeless or in care, lack of education, the extension of radiological involvement, cavitary disease, Charlson comorbidity index and some laboratory levels.

In-hospital mortality rate has been reported in various frequencies as 14% in Saudi Arabia in Taiwan (12.3%), China (18.9%), Korea (30.4%), the

Philippines (37.5%), and Pakistan (42.5%). Such differences in these countries might be because of the difference in the patient populations, ethnic backgrounds, and comorbidities (12-17). The mortality rate was found to be slightly lower in the present study. It may be caused by TB treatment model with higher directly observed therapy rate and payment-free treatment opportunity in Türkiye.

It is also important that TB mortality rates of long- term studies of ambulatory patients rather than hospitalized patients are generally lower. The overall long-term mortality rate of ambulatory TB patients is 0.14% in Poland and 6% in Saudi Arabia (18,19). This indicates that the highest mortality in TB patients occurs during the early phase of the disease or during hospitalization. When one-year mortality rates were evaluated, it was seen that most of the deaths occurred within the first month.

TB mostly affects elderly people although it might affect all age groups (20). Age has important roles in mortality related to TB (21). Various studies have reported older age as a predictor of TB mortality (14,17-19), which might be because of low immune response. Elderly patients had a higher in-hospital mortality in our study, which was in accordance with the data from the literature. When the CCI cut-off was 3.5, more deaths were seen in multivariate analysis

**Table:** 

**Table 5.** Univariate and multivariate logistic regression analysis of the variables predicting in-hospital mortality of tuberculosis						
		**Univariate analysis**			**Multivariate analysis**	
**Variables**	**OR**	**95%CI**	**p**	**OR**	**95%CI**	**p**
Age >48.5 years	6.48	4.11-10.2	**<0.001**	**2.90**	1.37-6.17	**0.006**
BMI <18.5 kg/m2	1.71	1.2-2.44	**0.003**	0.83	0.47-1.47	0.53
Hospital stay time <10.75 days	2.24	1.6-3.13	**<0.001**	**5.17**	2.81-9.30	**<0.001**
Unemployment	1.78	1.28-2.47	**0.001**	**2.74**	1.53-4.90	**0.001**
Uneducated	3.40	1.56-7.39	**0.002**	1.41	0.45-4.49	0.56
Smoker	1.43	1.02-2.01	**0.04**	1.77	0.98-3.22	0.06
Presence of comorbidity	6.43	4.09-10.13	**<0.001**	2.20	0.99-4.91	0.05
Chronic pulmonary disease	3.53	2.54-4.92	**<0.001**	0.95	0.51-1.75	0.86
Chronic renal failure	3.92	2.16-7.14	**<0.001**	2.55	0.25-26.25	0.43
Cardiovascular disease	2.83	1.93-4.16	**<0.001**	1.31	0.65-2.67	0.45
Malignancy	5.17	3.22-8.30	**<0.001**	**2.99**	1.36-6.58	**0.006**
Cerebrovascular disease	4.58	2.65-7.93	**<0.001**	2.04	0.76-5.46	0.16
Dementia	11.49	5.72-23.10	**<0.001**	4.11	1.43-11.75	**0.008**
Charlson comorbidity index >3.5	9.33	6.56 -13.26	**<0.001**	**3.11**	1.56-6.20	**0.001**
Extensive involvement	6.94	4.05-11.90	**<0.001**	**3.98**	1.48-10.76	**0.006**
Bilateral involvement	3.31	2.23-4.91	**<0.001**	1.23	0.59-2.54	0.58
Cavitary disease	1.83	1.28-2.64	**0.001**	**3.09**	1.67-5.69	**<0.001**
Protein <6.21 g/dL	8.50	5.98-12.10	**<0.001**	**2.35**	1.27-4.34	**0.007**
Albumin <3.03 g/dL	10.61	7.09-15.89	**<0.001**	**3.77**	1.86-7.62	**<0.001**
Hemoglobin <10.65 g/dL	3.342	2.408-4.638	**<0.001**	1.10	0.63-1.91	0.76
Lymphocytes <0.97/mm3	5.473	3.911-7.660	**<0.001**	**2.06**	1.03-4.09	**0.04**
Neutrophil >8.04/mm3	3.117	2.224-4.368	**<0.001**	**3.43**	1.83-6.40	**<0.001**
NLR >7.39	4.821	3.453-6.729	**<0.001**	0.76	0.37-1.55	0.443
BMI: Body mass index, AUC: Area under the curve, NLR: Neutrophil lymphocyte ratio, OR: Odds ratio, CI: Confidence interval.

(OR= 3.11 95% CI, 1.56-6.20, p< 0.001). It is more

common for elderly people to have multiple comorbidities, and this might change immune status delaying diagnosis [particularly in congestive heart failure (CHF), which can mimic TB symptoms]. Previous studies have reported that comorbidities (e.g. CHF, diabetes mellitus, renal failure, malignancy, and liver disease) are independent factors and significant predictors of TB-related mortality (12,17,22). Diabetes was not associated with mortality in this study. However, some studies have reported that diabetes increases the risk for early mortality while in the treatment of TB (6).

We revealed only comorbid malignancy to be an independent risk factor for in-hospital mortality in multivariate analysis (OR= 2.99, 95% CI 1.36-6.58, p= 0.006). Other studies have also reported that malignancy increases the mortality risk in TB (19-21).

Patients who have malignant tumors are immunocompromised because of local or systemic impacts of the disease and treatment regimens since these can impair the immune system and make them susceptible to TB development (23). TB might also have an unusual clinical manifestation, which makes its diagnosis more difficult, and contributes to delayed diagnosis and high mortality (24,25).

In the univariate analysis, protein, albumin, hemoglobin, and neutrophil to lymphocyte ratio were found to be significant, and neutrophil and albumin were significant predictors of mortality in the multivariate analysis. BMI was lower in the in-hospital death group, and overweight and obese patients were more in the surviving group. These might show that the patients who died had suppressed immune systems due to malnourishment, and for this reason, sicker upon initial presentation. Similar findings have also

been reported in a previous study (15). In general, although body weights were taken as nutritional status in studies, BMI, which reflects nutritional status better, was evaluated in the present study.

In the assessment of radiological features, the presence of a cavitary lesion and extensive pulmonary involvement were found as independent predictors in this study. Some radiographic characteristics were significant predictors of mortality (e.g. bilateral pulmonary involvement, pleural effusion, and miliary TB) in other studies. Such findings might result from diagnostic ambiguity in presentation and might cause delayed diagnosis and treatment initiation (9).

There were a total of 57 MDR cases in the study, and 8% of them died in hospital. Drug resistance was not found to be a risk factor for mortality in this study (which might be explained by the small sample size of the patients with MDR-TB). Similar to our study, it has been reported in some previous studies that drug resistance is not associated with mortality (9,14-17). However, MDR has been reported as a risk factor for TB-related mortality (20). According to the WHO data, 250.000 deaths were reported worldwide in 2015 because of MDR/RR-TB most of whom were from Asian countries (e.g. India, China, and the Russian Federation) (20). Another study reported a 21% mortality rate in MDR-TB (26).

Although HIV was presented as a condition increasing mortality, we could not reach such a conclusion since the number of HIV-positive patients was only six which corresponded to 0.5%. People who have HIV also have a greater risk of developing TB due to suppressed immune systems. According to the WHO data, HIV-positive people are 20-30 times more likely to develop active TB (20). HIV infection is an important risk factor regarding TB mortality (27). TB-HIV coinfection is associated with higher mortality rates, and a TB-HIV coinfection is taken an important predictor of TB mortality in general (28).

In the present study, the initial smear-negative or positive had no effects on mortality. However, it has been reported in other studies that smear-negative is an independent predictor of mortality. This was attributed to the delays in diagnosis in smear-negative patients (29). A recent retrospective cohort study in Brazil has found high mortality rates during hospitalization (16.1%), and negative sputum smear microscopy has been found to be an in-hospital mortality predictor (5). The mortality of smear-

negative patients has been found to be high in the study of Gaifer conducted in Oman (30).

It was also found in this study that homeless and unemployed patients were statistically higher in the deceased group. Literature has reported different results on this subject. Some reports that the mortality of communicable diseases other than tuberculosis is more related to economic status (31). It has been reported in some other studies that homelessness and unemployment increase the overall mortality of tuberculosis (32).

The first limitation of the study was that it had a retrospective and single-center fashion. This might not represent national mortality rates for TB patients who are hospitalized. Secondly, multiple factors that contributed to the cause of mortality might occur at the same time in TB patient mortality. For this reason, the reason for mortality might not be determined precisely, especially if autopsy is not performed. Another limitation is that the overall mortality rate after discharge or treatment provides information on TB population survival, but not all of these deaths may be the result of tuberculosis.

## CONCLUSION

Knowing the risk factors that increase in-hospital mortality is important for reducing early mortality. Early and rapid clinical management is required in pulmonary tuberculosis. For hospitalized tuberculosis patients, the first month of treatment is the most important and risky period in terms of mortality.

A cavitary or diffuse pulmonary disease, to be homeless, unemployed, uneducated, smoker or need care, low weight, hemoglobine, leucocyte, albumin levels, high Charlson comorbidity index, neutrophils and especially those with malignancy and chronic pulmonary disease are related with in-hospital mortality.

**Ethical Committee Approval:** This is a retrospective study, and local ethics approval SBÜ Dr. Suat Seren Training and Research Hospital was received with the ethics approval date and number 27-12-2017/ 8665. All procedures performed in studies involving human participants were carried out following the ethics standards of the institutional and/or national research committee and with the 1964 Helsinki declaration and its later amendments or comparable ethical standards.

## CONFLICT of INTEREST

The authors declare that they have no conflict of interest.

## AUTHORSHIP CONTRIBUTIONS

Concept/Design: MG, MAT, GP, DT, AEE Analysis/Interpretation: MG, MAT, ÖÖ Data acqusition: MAT, ÖÖ, FG, GA, OK Writing: MG, MAT, ÖÖ, GP

Clinical Revision: DT, AEE Final Approval: DT, AEE

## REFERENCES

1. *
  Chakaya J, Khan M, Ntoumi F, Aklillu E, Fatima R, Mwaba P, et al. Global tuberculosis report 2020 - Reflections on the global TB burden, treatment and prevention efforts. Int J Infect Dis 2021; 113(1): 7-12. https://doi.org/10.1016/j. ijid.2021.02.107*
2. *
  World Health Organization (WHO). Global tuberculosis report 2021. Geneva: World Health Organization; 2021.
  *
3. *
  T.C. Sağlık Bakanlığı Halk Sağlığı Genel Müdürlüğü. Tüberküloz tanı ve tedavi rehberi. 2. Baskı, Ankara, 2019.
  *
4. *
  Kim HJ, Lee C-H, Shin S, Lee JH, Kim YW, Chung HS, et al. The impact of nutritional defcit on mortality of in-patients with pulmonary tuberculosis. Int J Tuberc Lung Dis 2010; 14(1): 79-85.
  *
5. *
  Hansel NN, Merriman B, Haponik EF, Diette GB. Hospitalizations for tuberculosis in the United States in 2000: Predictors of in-hospital mortality. Chest 2004: 126(4): 1079-86. https://doi.org/10.1378/ chest.126.4.1079*
6. *
  Workneh MH, Bjune GA, Yimer SA. Diabetes mellitus is associated with increased mortality during tuberculosis treatment: A prospective cohort study among tuberculosis patients in South-Eastern Amahra Region, Ethiopia. Infect Dis Poverty 2016; 5(1): 22. https://doi.org/10.1186/ s40249-016-0115-z*
7. *
  Weiss MG, Sommerfeld J, Uplekar MW. Social and cultur- al dimensions of gender and tuberculosis. Int J Tuberc Lung Dis 2008; 12(7): 829-30.
  *
8. *
  Chung-Delgado K, Guillen-Bravo S, Revilla-Montag A, Bernabe-Ortiz A. Mortality among MDR-TB cases: Comparison with drug-susceptible tuberculosis and asso- ciated factors. PLoS One 2015; 10(3): e0119332. https:// doi.org/10.1371/journal.pone.0119332*
9. *
  World Health Organization (WHO). Definitions and reporting framework for tuberculosis - 2013 revision https://apps.who.int/iris/bitstr eam/han - dle/10665/79199/?sequence=1).
  *
10. *
  American Thoracic Society: National tuberculosis associa- tion of the USA. In: Diagnostic standards and classifica- tion of tuberculosis. New York. National Tuberculosis Association 1961.
  *
11. *
  Ashenafi S, Loreti MG, Bekele A, Aseffa G, Amogne W, Kassa E, et al. Inflammatory immune profiles associated with disease severity in pulmonary tuberculosis patients with moderate to severe clinical TB or anemia. Front Immunol 2023; 14: 1296501. https://doi.org/10.3389/ fimmu.2023.1296501*
12. *
  Aljohaney AA. Mortality of patients hospitalized for active tuberculosis in King Abdulaziz University Hospital, Jeddah, Saudi Arabia. Saudi Med J 2018; 39(3): 267-72. https://doi.org/10.15537/smj.2018.3.22280*
13. *
  Lin CH, Lin CJ, Kuo YW, Wang JY, Hsu CL, Chen JM, et al. Tuberculosis mortality: Patient characteristics and causes. BMC Infect Dis 2014; 14: 5. https://doi.org/10.1186/1471- 2334-14-5*
14. *
  Lui G, Wong RY, Li F, Lee MK, Lai RW, Li TC, et al. High mortality in adults hospitalized for active tuberculosis in a low HIV prevalence setting. PLoS One 2014; 9: e92077. https://doi.org/10.1371/journal.pone.0092077*
15. *
  Kim CW, Kim SH, Lee SN, Lee SJ, Lee MK, Lee JH, et al. Risk factors related with mortality in patient with pulmonary tuberculosis. Tuberc Respir Dis 2012; 73: 38-47. https:// doi.org/10.4046/trd.2012.73.1.38*
16. *
  Shimazaki T, Marte SD, Saludar NR, Dimaano EM, Salva EP, Ariyoshi K, et al. Risk factors for death among hospital- ised tuberculosis patients in poor urban areas in Manila, The Philippines. Int J Tuberc Lung Dis 2013; 17: 1420-6. https://doi.org/10.5588/ijtld.12.0848*
17. *
  Haque G, Kumar A, Saifuddin F, Ismail S, Rizvi N, Ghazal S, et al. Prognostic factors in tuberculosis related mortali- ties in hospitalized patients. Tuberc Res Treat 2014; 624671. https://doi.org/10.1155/2014/624671*
18. *
  Abouzeid MS, Al Hakeem RF, Memish ZA. Mortality among tuberculosis patients in Saudi Arabia (2001-2010). Ann Saudi Med 2013; 33: 247-52. https://doi. org/10.5144/0256-4947.2013.247*
19. *
  Koseła MK. Tuberculosis in Poland in 2014. Przegl Epidemiol 2016; 70: 261-72.
  *
20. *
  World Health Organization (WHO). Global tuberculosis report 2016.
  *
21. *
  Heunis JC, Kigozi NG, Chikobvu P, Botha S, van Rensburg HD. Risk factors for mortality in TB patients: A 10-year electronic record review in a South African province. BMC Public Health 2017; 17: 38. https://doi.org/10.1186/ s12889-016-3972-2*
22. *
  Walpola HC, Siskind V, Patel AM, Konstantinos A, Derhy P. Tuberculosis-related deaths in Queensland, Australia, 1989-1998: Characteristics and risk factors. Int J Tuberc Lung Dis 2003; 7: 742-50.
  *
23. *
  Kamboj M, Sepkowitz KA. The risk of tuberculosis in patients with cancer. Clin Infect Dis 2006; 42(11): 1592- 5. https://doi.org/10.1086/503917*
24. *
  Kim HR, Hwang SS, Ro YK, Jeon CH, Ha DY, Park SJ, et al. Solid-organ malignancy as a risk factor for tuberculosis. Respirology 2008; 13(3): 413-9. https://doi.org/10.1111/ j.1440-1843.2008.01282.x*
25. *
  Silva FA, Matos JO, de QMF, Nucci M. Risk factors for and attributable mortality from tuberculosis in patients with hematologic malignances. Haematologica 2005; 90(8): 1110-5.
  *
26. *
  Salinas JL, Armstrong LR, Silk BJ, Haddad MB, Cegielski JP. Factors associated with all-cause mortality among patients with multidrug-resistant tuberculosis-United States, 1993- 2013. Clin Infect Dis 2017; 65: 1924-6. https://doi. org/10.1093/cid/cix667*
27. *
  Field N, Lim MS, Murray J, Dowdeswell RJ, Glynn JR, Sonnenberg P, et al. Timing, rates, and causes of death in a large South African tuberculosis programme. BMC Infect Dis 2014; 14: 3858. https://doi.org/10.1186/s12879- 014-0679-9*
28. *
  Mabunda TE, Ramalivhana NJ, Dambisya YM. Mortality associated with tuberculosis/HIV co-infection among patients on TB treatment in the Limpopo province, South Africa. Afr Health Sci 2014; 14: 849-54. https://doi. org/10.4314/ahs.v14i4.12*
29. *
  Rao VK, Iademarco EP, Fraser VJ, Kollef MH. Te impact of comorbidity on mortality following in-hospital diagnosis of tuberculosis. Chest 1998; 114(5), 1244-52. https://doi. org/10.1378/chest.114.5.1244*
30. *
  Gaifer ZA. Risk factors for tuberculosis mortality in a tertia- ry care center in Oman, 2006-2016. Int J Mycobacteriol 2017; 6(4): 356-9. https://doi.org/10.4103/ijmy. ijmy_123_17*
31. *
  Zilidis C, Papagiannis D, Kyriakopoulou Z. Did economic crisis affect mortality due to infectious diseases? Trends of infectious diseases mortality in Greece before and after economic crisis. Cureus 2021;13(2): e13621. https://doi. org/10.7759/cureus.13621*
32. *
  Hannah HA, Miramontes R, Gandhi NR. Sociodemographic and clinical risk factors associated with tuberculosis mortality in the United States, 2009- 2013. Public Health Rep 2017; 132(3): 366-75. https:// doi.org/10.1177/0033354917698117*

